# Wharton’s jelly mesenchymal stem cells embedded in PF-127 hydrogel plus sodium ascorbyl phosphate combination promote diabetic wound healing in type 2 diabetic rat

**DOI:** 10.1186/s13287-021-02626-w

**Published:** 2021-10-30

**Authors:** Yiren Jiao, Xiaolin Chen, Yongxia Niu, Sunxing Huang, Jingwen Wang, Mingxun Luo, Guang Shi, Junjiu Huang

**Affiliations:** 1 MOE Key Laboratory of Gene Function and Regulation, State Key Laboratory of Biocontrol, School of Life Sciences, Sun Yat-Sen University, Guangzhou, 510275 China; 2 Key Laboratory of Reproductive Medicine of Guangdong Province, The First Affiliated Hospital and School of Life Sciences, Sun Yat-Sen University, Guangzhou, 510275 China

**Keywords:** Diabetic wound healing, Wharton’s jelly mesenchymal stem cells, PF-127 hydrogel, Sodium ascorbyl phosphate, Rat

## Abstract

**Background:**

Diabetic cutaneous ulcers (DCU) are a complication of diabetes with diabetic foot ulcers being the most common, and the wounds are difficult to heal, increasing the risk of bacterial infection. Cell-based therapy utilizing mesenchymal stem cells (MSCs) is currently being investigated as a therapeutic avenue for both chronic diabetic ulcers and severe burns. Wharton’s jelly mesenchymal stem cell (WJMSC) with PF-127 hydrogel and sodium ascorbyl phosphate (SAP) improved skin wound healing in mice. Whether this combination strategy is helpful to diabetic ulcers wound healing remains to be explored.

**Methods:**

Firstly, the WJMSCs embedded in PF-127 and SAP combination were transplanted onto excisional cutaneous wound bed in type 2 diabetic Sprague Dawley (SD) rats. Two weeks after transplantation, the skin tissue was collected for histological and immunohistochemical analysis. Further, overexpressing-EGFP WJMSCs were performed to investigate cell engraftment in the diabetic cutaneous ulcer. The apoptosis of WJMSCs which encapsulated with combination of PF-127 and SAP was detected by TUNEL fluorescence assay and RT-PCR in vitro. And the mitochondrial damage induced by oxidative stress assessed by MitoTracker and CMH2DCFDA fluorescence assay.

**Results:**

In diabetic cutaneous wound rat model, PF-127 plus SAP-encapsulated WJMSCs transplantation promoted diabetic wound healing, indicating improving dermis regeneration and collagen deposition. In diabetic wound healing, less pro-inflammatory M1 macrophages, more anti-inflammatory M2 tissue-healing macrophages, and neovascularization were observed in PF-127 + SAP + WJMSCs group compared with other groups. SAP supplementation alleviated the apoptosis ratio of WJMSCs embedded in the PF-127 in vitro and promoted cell survival in vivo.

**Conclusion:**

PF-127 plus SAP combination facilitates WJMSCs-mediated diabetic wound healing in rat through promoting cell survival, the macrophage transformation, and angiogenesis. Our findings may potentially provide a helpful therapeutic strategy for patients with diabetic cutaneous ulcer.

**Supplementary Information:**

The online version contains supplementary material available at 10.1186/s13287-021-02626-w.

## Introduction

Patients with diabetes worldwide have gradually increased year by year and attracted greater attention [1]. Diabetic foot ulcers (DFU) are a complication of diabetes with being the most common, and the wounds are difficult to heal, increasing the risk of bacterial infection and amputation [2]. Due to the high glucose in the diabetes patients, the immune response is insufficient caused by the defective leukocyte and decreased neovascularization. In addition, dermal cells differentiation decreased [3]. The conventional therapeutic alternatives including drug therapy, physical therapy, and surgical treatment, cannot treat DFU effectively accompanied by delayed healing and scarring. Given that mesenchymal stem cells (MSCs) promote wound healing in acute or chronic cutaneous damage, MSCs-based cell therapy has a potential therapeutic avenue for wounds healing in DFU [4, 5].

MSCs participated into the tissue reparation and regeneration through paracrine effects and highly plastic immunoregulation [6, 7]. Wharton’s jelly-derived mesenchymal stem cells (WJMSCs) isolated from the fetus umbilical cord have the higher proliferative ability, stronger immunomodulatory effects, easier solution, fewer ethical issues, and more safely than other resources-derived MSCs [8]. WJMSCs may promote the epithelialization through increasing the release of cytokeratin 19 and the formation of extracellular matrix formation in diabetic Sprague Dawley (SD) rats [9]. And WJMSCs transplantation after angioplasty promotes the new vessels formation and ulcers healing of DFU patients [10]. However, hyperglycemia in DFU severely reduces the therapeutic effect of MSCs, which affect cell survival and engraftment [11, 12]. Thus, MSCs-derived wound healing in DFU needs to be improved to solve bottleneck problems, such as poor engraftment, short retention, and low survival.

Scaffold-based cell applications enhanced the viability and paracrine of MSCs [13]. WJMSCs carried with the biological scaffold can significantly improve the average healing rate of chronic wound [14]. Pluronic F-127 (PF-127) as an injectable, biodegradable, and temperature-sensitive scaffold, has been approved by Food and Drug Administration (FDA) for clinical application of stem cells [15, 16]. Combination of PF-127 and exosomes derived from WJMSCs can significantly promote wound healing in diabetic rats [17]. However, PF-127 has a certain cytotoxicity on encapsulated cells [18]. In order to reduce the toxicity of PF-127 to WJMSC, recent studies have showed that adding membrane-stabilizing agent steroid hydrocortisone and platelet-rich plasma can significantly increase MSCs survival in PF-127 [19, 20]. In addition, our previous studies have shown that sodium ascorbyl phosphate (SAP) can greatly enhance the viability of WJMSCs carried by PF127 and promote skin wound healing in mice [21]. SAP is the sodium salt of ascorbic acid 2-phosphate which has high stability even on after long-term exposure to reactive oxygen species as well as aqueous solution [22]. Based on its role in promoting wound healing by WJMSCs, whether the strategy can promote wound healing of DFU remains to be further explored.

In this study, we used high-fat diet feeding combination with streptozotocin (STZ) injection to induce diabetic model in rat and then constructed the full-thickness cutaneous wound to simulate diabetic wound. WJMSCs encapsulated with PF-127 and SAP were transplanted into the wounds of diabetic ulcer in rats, and we found it could promote diabetic wound healing. And the dermis regeneration, the macrophage transformation, and neovascularization were also observed in diabetic rats. This study will provide a new MSCs-derived therapeutic strategy for diabetic wound healing.

## Materials and methods

### Culture of WJMSCs

The WJMSCs were isolated from human umbilical cord and identified as our previous study report [21]. The WJMSCs were seeded in 90% DMEM/F12 media with 10% fetal bovine serum (FBS) and 1% penicillin/streptomycin (Gibco, Grand Island, NY) at 37 °C in an incubator with 5% CO_2_. The culture medium was changed every 3 days.

### Cell viability assay

WJMSCs were seeded at a density of 1 × 10^5^/mL with different encapsulation conditions in 96-well plate and cultured at 37 °C and 5% CO_2_ for 24 h. Cell viability was evaluated by Cell Counting Kit-8 (CK04, Dojindo, Japan). Ten microliter CCK-8 reagent was added into medium each well and incubated for 2 h. Finally, the absorbance at 450 nm was measured by microplate reader (VICTOR™ X5, PerkinElmer, USA).

### Live/Dead assay

Live/Dead™ Cell Imaging Kit (R37601, Invitrogen, USA) was used to identify the survival of WJMSCs encapsulated with PF-127 hydrogel. Following the manufactures’ instruction, 5 × 10^4^ WJMSCs per well with different encapsulated conditions were seeded into 12 well-plate for 24 h. The component A (live green) was transferred into the component B (dead red) to produce the working solution. Then, equal volume of working solution was added into each well and incubated at 25 °C in dark for 15 min. The fluorescence images were captured by inverted fluorescence microscope (Axio observer A1, Zeiss, Germany).

### Cell proliferation assay

The cell proliferation ability was assessed by Cell Light™ EdU Kit (C10310, Ribo Biotech Company, China) following the manufacturer’s instructions. WJMSCs were seeded at a density of 5 × 10^4^/well with different encapsulation conditions in 48-well plate for 24 h. Then, 50 μM EdU was added to complete medium of each well for another 2 h. After fixation and permeabilization, Apollo and DAPI staining were conducted. EdU-positive WJMSCs were observed and captured using an inverted fluorescence microscope (Axio observer A1, Zeiss, Germany). Fluorescent images from WJMSCs were analyzed using Image Pro Plus (Media Cybernetics, Inc., Silver Spring, MD, USA).

### MitoTracker assay

The mitochondria of WJMSCs with different encapsulation conditions were tracked by MitoTracker® Mitochondrion (M7512, Thermo Fisher Scientific, USA). WJMSCs were seeded with different encapsulation conditions in 24-well plate for 24 h. WJMSCs were incubated with prewarmed (37 °C) staining solution containing MitoTracker® probe for 15 min and, then, replaced the staining solution with fresh prewarmed complete media. Fluorescent images of MitoTracker were observed and captured using an inverted fluorescence microscope (Axio observer A1, Zeiss, Germany).

### Reactive oxygen species (ROS) measurement

ROS production in WJMSCs with different encapsulation conditions was detected by CMH2DCFDA (C6827, Thermo Fisher Scientific, USA) fluorescence assay. WJMSCs were seeded with different encapsulation conditions in 24-well plate for 24 h. WJMSCs were incubated in complete medium with 5 μΜ CM-H2DCFDA for 20 min at 37 °C in 5% CO_2_ atmosphere and, then, washed the WJMSC with PBS for three time and added the new complete medium into the plate well. Fluorescent images of ROS-positive WJMSCs were observed and captured using an inverted fluorescence microscope (Axio observer A1, Zeiss, Germany).

### TUNEL assay

Cell apoptosis in WJMSCs with different encapsulation conditions was estimated by terminal deoxynucleotidyl transferase-mediated dUTP nick end-labeling (TUNEL) assay. In brief, WJMSCs with different encapsulation conditions were fixed with 4% paraformaldehyde and blocked with 3% H_2_O_2_ at room temperature. After being permeabilize in 0.1% TritonX-100 for 2 min at 4 °C, the cells were treated by TUNEL detection Kit (G002, Nanjing Jiancheng Bioengineering Institute, China). The cell nuclei were stained with 4’, 6-diamidino-2-phenylindole (DAPI). TUNEL-positive WJMSCs were observed and captured using an inverted fluorescence microscope (Axio observer A1, Zeiss, Germany).

### Establishment of WJMSCs stably expressing EGFP

We firstly constructed the EGFP overexpressing lentiviral expression vector pLenti-CAG-EGFP-IRES-Puro. Lentivirus was harvested and concentrated after co-transfected the HEK293T cells with pLenti-CAG-EGFP-IRES-Puro lentiviral packaging vectors mix, pMD2.G, and psPAX2 by PEI (306,185, Sigma, USA). The P3 WJMSCs were incubated onto the 6-well plate and were infected with the lentivirus particles and 4 μg/mL polybrene (TR1003, Sigma, USA). At last, 1 mg/mL Puromycin was added into the culture medium to screen the EGFP-positive WJMSCs for 3 days. After that, OE-EGFP WJMSCs were re-cultured with complete medium and were further verified with immunofluorescence assay and western blot.

### Animals

Four-week-old male Sprague Dawley rats were purchased from the Animal Center of East Campus of Sun Yat-Sen University (Guangzhou, China). All experiments involving animals were performed in accordance with guidelines approved by the Institutional Animal Care and Use Committee of Sun Yat-Sen University, P.R. China.

### Establishment of cutaneous wounds in diabetic rats

Four-week-old healthy male Sprague Dawley (SD) rats (weight: 60–80 g) were housed at a constant temperature and humidity. All SD rats were adaptively feed under normal condition for 1 week. After that, we construct the diabetic rat model according to previous report [17, 23]. Briefly, high-glucose and high-fat diet was used to feed the SD rats for 4 weeks before streptozotocin (STZ) injection. And then, SD rats were intraperitoneally injected with 40 mg/kg STZ (S0130-50MG; Sigma-Aldrich, St. Louis, MO, USA), which was prepared by 0.1 mM sodium citrate buffer solution. The fasting blood glucose level was measured by an offline blood glucose monitoring system (Accu-Chek Advantage strips; Roche Diagnostics, Mannheim, Germany) at day 3 and day 7 post-injection. SD rats with a blood glucose level between 11.2 and 16.7 mM continuously for 7 days were considered type 2 diabetic rats.

Diabetic SD rats were anesthetized, disinfected, and sheared before ulcer wound construction. Skin biopsy device was used to form an 8-mm circular full-thickness skin wound on both back of each rat. All diabetic rats were divided into 8 groups, including: PBS, 400 μM SAP, PF-127, PF-127 + 400 μM SAP, WJMSCs, WJMSCs + 400 μM SAP, WJMSCs + PF-127, WJMSCs + PF-127 + 400 μM SAP. Total 50 μL PF-127 + 400 μM SAP combination encapsulated 2 × 10^6^ WJMSCs (passage 6) or other combinations were transplanted onto the wound site and then covered with IV3000 transparent dressing to avoid infection. The skin wound of each rat was investigated and captured the representative images at 0, 3, 7, 10, 14 days after transplantation. The unhealed wound area was measured by ImageJ software as follows: unhealed wound area (%) = Wr/Wi × 100%, where Wi is the initial wound area at day 0, while Wr is the residual wound area at day 7 and day 14 post-transplantation.

### PF-127 hydrogel plus SAP combination preparation and WJMSCs encapsulation

20% (w/v) PF-127 solution was dissolved in DMEM-F12 medium at 4 °C, then filtered with 0.22-μm filter (Millipore, USA), and stored at 4 °C. 400 μM SAP (49,752, Sigma, USA) was added into the 20% PF-127 solution to form the PF-127 hydrogel plus SAP combination. For in vitro PF-127 hydrogel cytotoxicity test, PF-127 solution with 400 μM SAP-encapsulated WJMSCs and incubated in 37 °C incubator (5% CO_2_) for 15 min to make gel formation. After that, the complete culture medium was added to cover the gel and co-culture for another 24 h.

### Histological analysis

At the day 14 post-transplantation, the SD rats were killed for histological analysis. Briefly, the ulcer wound bed together with surrounding tissue was excised and underwent following standard procedures including 4% paraformaldehyde fixation, gradual dehydration, and paraffin embedding. The embedded tissues were then sliced into 5-μm-thick sections in the direction of hair flow, which were further stained with Hematoxylin and Eosin Staining Kit (G1120, Solarbio, China) and Masson Trichrome Staining Kit (D026, Nanjing Jiancheng Bioengineering Institute, China). ImageJ software was used to measure the thickness of dermis, scar width, and the total number of hair follicles.

### Immunohistochemical analysis

Paraffin sections of each group were undergone antigen retrieval with sodium citrate buffer and blocked by 3% goat serum (16210064, Gibco, USA) for 1 h. Diluted primary antibodies (anti-CD31 antibody (Abcam, ab28364); anti-Ki-67 antibody (Cell Signaling Technology, 12202T); anti-CD163 antibody (Abcam, ab182422); anti-CD86 antibody (Cell Signaling Technology, 19589S)) with blocking buffer were used to incubate at 4 °C overnight. After washing with PBS, the cell and tissue staining kit (CTS005, Anti-Rabbit HRP-DAB System, R&D Systems) was used to detect the staining. Images were taken by an inverted fluorescence microscope (Axio observer A1, Zeiss, Germany) and analyzed by ImageJ software.

### Tracking of OE-EGFP WJMSCs in vivo

To track the distribution of transplanted OE-EGFP WJMSCs in vivo, the samples were collected from the diabetic ulcer cutaneous tissue at 24 h and 72 h post-transplantation. Cryosections were prepared and counterstained with DAPI for 5 min. After that, the sections were observed and captured with an inverted fluorescence microscope (Axio observer A1, Zeiss, Germany). The survival OE-EGFP WJMSCs were counted by ImageJ software.

### Immunofluorescence staining

The frozen sections of diabetic rat skin were permeated with 0.5% Triton-100 and then sealed with sealing solution at room temperature for 1 h. The primary antibody (Monoclonal ANTI-FLAG® M2-Cy3, A9594, Sigma, Germany) was incubated overnight and stained with DAPI for 10 min. After that, the sections were observed and captured with an inverted fluorescence microscope (Axio observer A1, Zeiss, Germany).

### RNA extraction and qPCR analysis

The methods used for the RNA extraction and PCR analysis have been described previously [24]. Briefly, total RNAs were extracted from WJMSCs using TRIzol reagent (Invitrogen) according to the manufacturer’s instructions. Total RNAs (1 μg) were reverse transcribed to cDNA using PrimeScript™ RT reagent Kit with gDNA Eraser (RR047A, TaKaRa, Otsu, Shiga, Japan). TB Green® Premix Ex Taq™ (Tli RNaseH Plus) (RR820A, TaKaRa, Otsu, Shiga, Japan) were used for qPCR. The PCRs were carried out on a CFX96TM Optical Reaction Module (Bio-Rad, Hercules, CA, USA). The relative expression of mRNAs was normalized with β-actin levels using the 2^−ΔΔCt^ method. Primers used for qPCR are shown in Additional file 1: Table 1.

### Western blot assay

Methods used for Western blot assay have been described previously [25]. WJMSCs lysed in RIPA buffer (Beyotime, P0013C) containing a protease inhibitor cocktail (Beyotime, P1005). About 30 μg protein lysates were separated using 10% SDS-PAGE and then transferred to PVDF membranes (Bio-Rad, 1,620,177) by using a Trans-Blot Turbo Transfer System (Bio-Rad). The membranes were blocked with 5% skim milk buffer for 2 h at room temperature and incubated with different diluted antibodies at 4 °C overnight. The membranes were then washed with Tris-buffered saline containing 0.05% Tween-20 before incubation with HRP-conjugated secondary antibodies for 2 h. Finally, the immunoreactive protein bands were detected and imaged by enhanced chemiluminescence (ECL, FDbio Science, FD8030) using the Image Lab system (Bio-Rad). The antibodies used in this study are listed in Additional file 1: Table 2. The band intensities were quantified with ImageJ and normalized to those of β-actin.

### Statistical analysis

All the data were demonstrated as mean ± standard error of mean (SEM). The assumptions of normality of data and homogeneity of variances between the groups were analyzed by SPSS. Significant differences between treatment groups were determined by one-way ANOVA (SPSS 18.0, Chicago, IL, USA). *p* < 0.05 was considered statistically significant (*p < 0.05, **p < 0.01, ***p < 0.001; ns, no significance).

## Results

### PF-127 plus SAP combination facilitates WJMSCs-mediated diabetic cutaneous ulcer wound healing

**Fig. 1:**
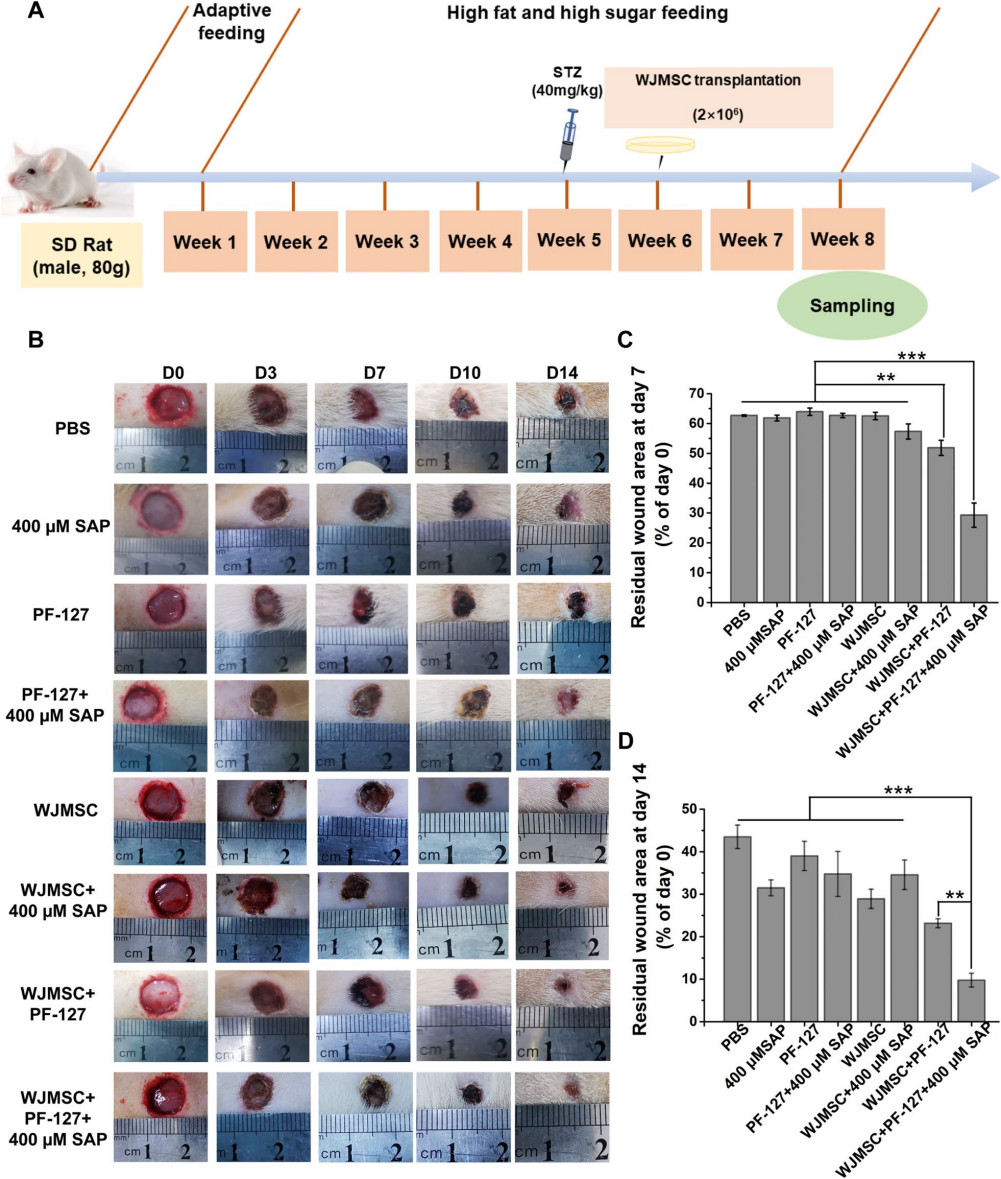
PF-127 plus SAP combination facilitates WJMSC-mediated diabetic wound healing.** a** Schematic of in vivo wound-healing assay after WJMSCs transplantation in STZ-induced diabetic SD rats. **b** Representative images of wounded skin were showed at 0, 3, 7, 10, 14 days post-transplantation. **c** The percentage of residual wound area of each group was analyzed at day 7 after transplantation. Error bars represent mean ± SEM; n = 8 independent experiments. Significance was determined using one-way ANOVA. **p < 0.01, ***p < 0.001. **d** The percentage of residual wound area of each group was analyzed at day 14 after transplantation. Error bars represent mean ± SEM; n = 8 independent experiments. Significance was determined using one-way ANOVA. **p < 0.01, ***p < 0.001

Our previous study found that PF-127 plus SAP improves WJMSCs-mediated acute skin wound healing in mice [21]. To investigate whether the combination of PF-127 and SAP promotes the WJMSCs-mediated diabetic cutaneous ulcer healing in vivo, we constructed the diabetic cutaneous ulcer model in rats. The body weight of diabetic SD rats decreased significantly after STZ infection (Additional file 1: Fig. S1A). Meanwhile, the fasting blood sugar of diabetic rats increased after STZ injection (Additional file 1: Fig. S1B). And then, WJMSCs embedded in PF-127 + SAP combination were transplanted into the diabetic cutaneous ulcer wound (Fig. 1a). Comparing with the control groups, PF-127 + SAP + WJMSCs group had the lowest residual wound area at day 7 (29.30 ± 4.08%) after transplantation (*p* < 0.001) (PBS, 62.70 ± 0.26%; SAP, 61.84 ± 0.96%; PF-127, 63.97 ± 1.24%; PF-127 + SAP, 62.71 ± 0.74%; WJMSCs, 62.53 ± 1.22; WJMSCs + SAP, 57.32 ± 2.55%; WJMSCs + PF-127, 51.86 ± 2.54%) (Fig. 1b, c). Similarly, at day 14 after transplantation, the diabetic cutaneous ulcer wound in the PF-127 + SAP + WJMSCs group was almost completely healed (9.73 ± 1.63%), whereas other groups still had visible unhealed wounds (*p* < 0.01) (PBS, 43.48 ± 2.75%; SAP, 31.45 ± 1.86%; PF-127, 38.98 ± 3.45%; PF-127 + SAP, 34.72 ± 5.30%; WJMSCs, 28.86 ± 2.26; WJMSCs + SAP, 34.53 ± 3.49%; WJMSCs + PF-127, 23.14 ± 1.05%) (Fig. 1b, d). These results implied that transplantation of WJMSCs encapsulated with PF-127 plus SAP can accelerate the diabetic wound healing in vivo.

### Improvement of dermis regeneration capacity and collagen deposition in diabetic wound upon WJMSC/PF127/SAP transplantation

**Fig. 2:**
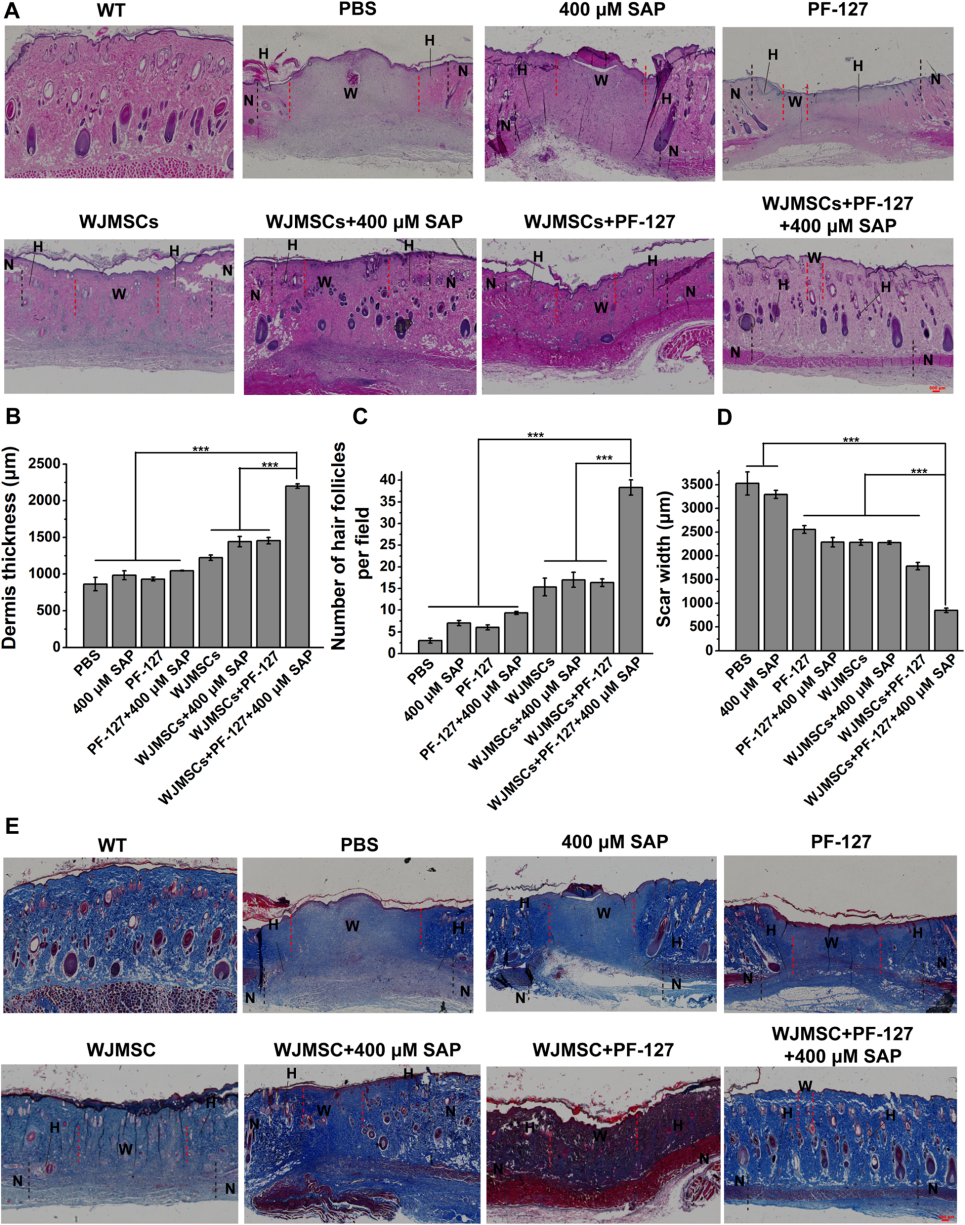
Histology analysis of the dermis regeneration and collagen deposition.** a** Hematoxylin and eosin staining in diabetic wound bed and surrounding normal tissues were analyzed at day 14 after transplantation. Scar bar: 500 μm. **b** The quantitative data of the dermis thickness in each group were showed at day 14 after transplantation. Error bars represent mean ± SEM; n = 8 independent experiments. Significance was determined using one-way ANOVA. ***p < 0.001. **c** The quantitative data of the number of newborn hair follicles in each group at day 14 post-transplantation per field. Error bars represent mean ± SEM; n = 8 independent experiments. Significance was determined using one-way ANOVA. ***p < 0.001. **d** The quantitative data of the scar width in each group were showed at day 14 post-transplantation. Error bars represent mean ± SEM; n = 8 independent experiments. Significance was determined using one-way ANOVA. ***p < 0.001. **e** Masson’s trichrome staining of diabetic wound bed and surrounding normal tissues was showed at day 14 after transplantation. Scar bar: 500 μm. N, normal skin tissue, shown on both sides of the black imaginary line; H, healed skin tissue, shown between black and red imaginary lines; W, wound bed, unhealed skin tissue, shown between the red imaginary lines

Regeneration of the dermis requires the migration of basal keratinocytes and the reconstruction of three-dimensional collagen structure mediated by fibroblasts and myofibroblasts [26, 27]. In order to investigate the therapeutic effect of WJMSCs encapsulated with PF-127 and SAP on the diabetic wound healing, we further observed the dermis regeneration and collagen deposition by histology analysis. The results showed that the dermis thickness in the WJMSCs + PF-127 + SAP group (2199.34 ± 30.14 μm) was thicker than other groups (PBS, 862.07 ± 92.03; SAP, 983.81 ± 60.26; PF-127, 931.15 ± 24.19; PF-127 + SAP, 1044.64 ± 5.15; WJMSCs, 1223.23 ± 38.19; WJMSCs + SAP, 1443.56 ± 70.86; WJMSCs + PF-127, 1455.28 ± 43.49 μm) (Fig. 2a, b) (*p* < 0.001). The newborn hair follicles in the WJMSCs + PF-127 + SAP group (38.33 ± 1.76 per field) were also more than other groups (PBS, 3.00 ± 0.58; SAP, 7.00 ± 0.57; PF-127, 6.00 ± 0.58; PF-127 + SAP, 9.33 ± 0.33; WJMSCs, 15.33 ± 2.03; WJMSCs + SAP, 17.00 ± 1.73; WJMSCs + PF-127, 16.33 ± 0.88 per field) (Fig. 2a, c) (*p* < 0.001). Conversely, the scar width in the WJMSCs + PF-127 + SAP group (850.08 ± 45.71 μm) was narrower than other groups (PBS, 3527.01 ± 244.19; SAP, 3295.36 ± 86.57; PF-127, 2554.13 ± 82.40; PF-127 + SAP, 2287.19 ± 100.17; WJMSCs, 2282.30 ± 58.01; WJMSCs + SAP, 2277.01 ± 35.48; WJMSCs + PF-127, 1782.22 ± 78.12 μm) (Fig. 2a, d) (*p* < 0.001). These results demonstrated that WJMSC + PF127 + SAP transplantation promotes the dermis regeneration. In addition, the collagen disposition in the ulcer wound was detected by Masson’s trichrome staining. The results showed that the disposition of collagen fibers in the WJMSCs + PF-127 + SAP group was significantly more than other groups (Fig. 2a, e). These results indicated that PF-127 plus SAP-encapsulated WJMSCs transplantation was indeed helpful to wound healing of diabetic ulcer.

### WJMSC/PF127/SAP transplantation facilitates macrophage transformation and re-epithelialization

**Fig. 3:**
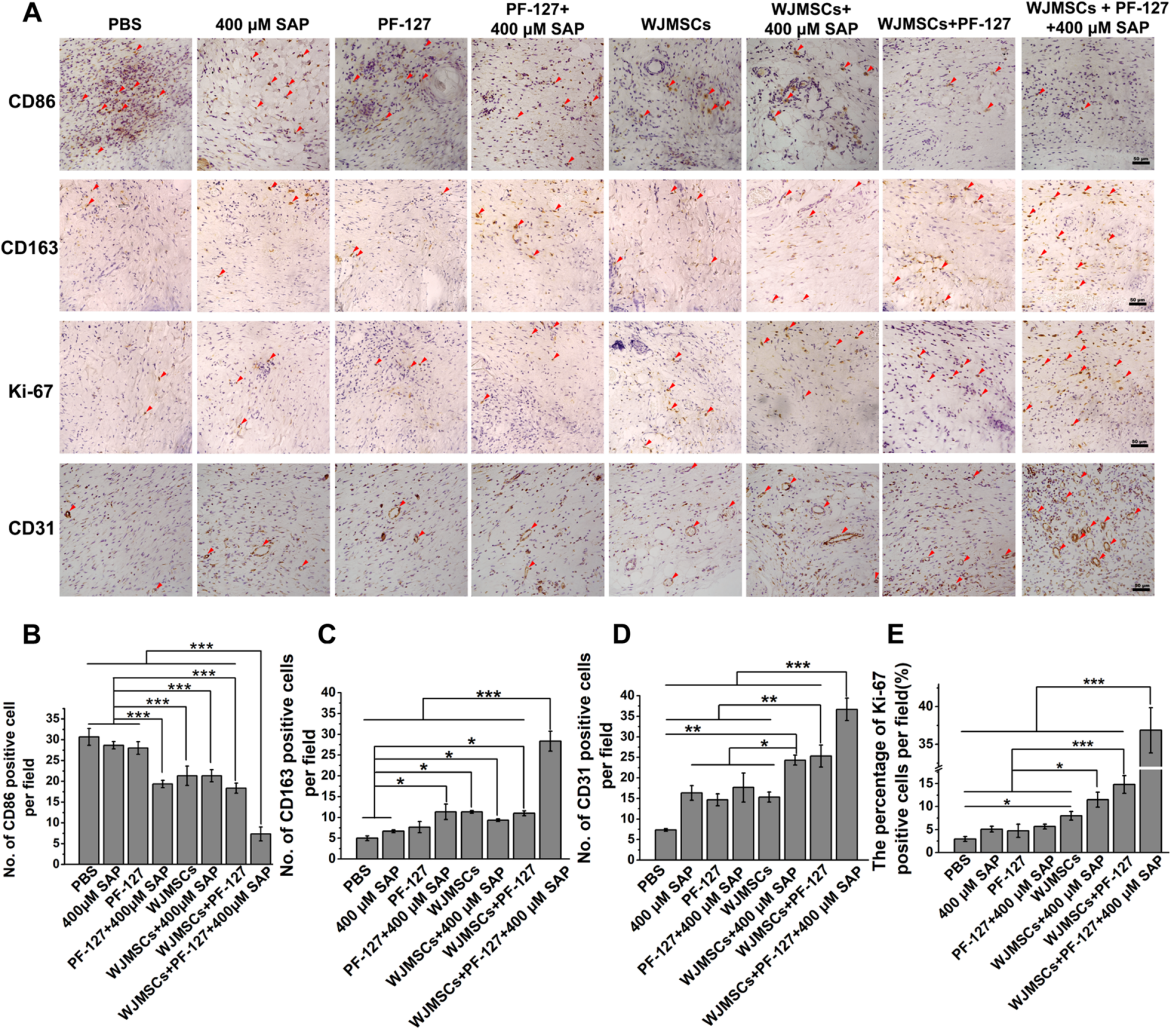
Immunohistochemistry identifies the macrophage transformation, cell proliferation, and neovascularization.** a** Immunohistochemical staining with anti-CD86, CD163, Ki-67, and CD31 antibodies on diabetic wound in each group was performed and analyzed at 14 days post-transplantation. Scar bar: 50 μm. **b, c** The quantitative data of the total number of CD86-positive M1 macrophages (**b**) and CD163-positive M2 macrophages (**c**) per field were analyzed. Error bars represent mean ± SEM; n = 3 independent experiments. Significance was determined using one-way ANOVA. *p < 0.05, ***p < 0.001. **d** The quantitative data of the percentage of Ki-67-positive proliferating cells per field were analyzed. Error bars represent mean ± SEM; n = 3 independent experiments. Significance was determined using one-way ANOVA. *p < 0.05, ***p < 0.001. **e** The quantitative data of the total number of CD31-positive newly formed blood vessels were analyzed. Error bars represent mean ± SEM; n = 3 independent experiments. Significance was determined using one-way ANOVA. *p < 0.05, **p < 0.01, ***p < 0.001

Cutaneous wound healing undergoes hemostasis, inflammatory, cell proliferation and re-epithelialization, and remodeling [28]. Macrophage temporally regulates polarization during skin wound healing to ameliorate the inflammatory environment of wound [29, 30]. Activated M1 pro-inflammatory macrophages marked by CD86 are responsible for combating infections; furthermore, activated M2 macrophages marked by CD163 are connected with tissue remodeling [31]. Uncontrolled or prolonged inflammatory responses lead to disruption of subsequent stages of wound healing, ultimately leading to the development of non-healing diabetic wounds [32, 33]. To explore the role of inflammatory regulation on diabetic wound after PF-127/SAP combination encapsulated WJMSCs transplantation, we evaluated the M1 and M2 macrophages formation in the diabetic wound via immunohistochemical staining with anti-CD86 and anti-CD163 antibodies, respectively. The results showed that the number of CD86-positive M1 macrophages in WJMSCs + PF-127 + 400 μM SAP group (7.33 ± 1.67 per field) was lower than others groups (PBS, 30.67 ± 2.03; SAP, 28.67 ± 0.88; PF-127, 28.00 ± 1.53; PF-127 + SAP, 19.33 ± 0.88; WJMSCs, 21.33 ± 2.33; WJMSCs + SAP, 20.33 ± 1.45; WJMSCs + PF-127, 18.33 ± 1.20 per field) (Fig. 3a, b) (*p* < 0.001), while the number of CD163-positive M2 macrophages (28.33 ± 2.40 per field) was more than other groups (Fig. 3a, c) (*p* < 0.001) (PBS, 5.00 ± 0.58; SAP, 6.67 ± 0.33; PF-127, 7.67 ± 1.33; PF-127 + SAP, 11.33 ± 1.86; WJMSCs, 11.32 ± 0.33; WJMSCs + SAP, 9.33 ± 0.33; WJMSCs + PF-127, 11.00 ± 0.58 per field). These results indicated that WJMSCs + PF-127 + SAP transplantation facilitated macrophages transformation from proinflammatory M1 type to anti-inflammatory M2 type.

Re-epithelialization initiates approximately 16–24 h after injury, accompanied by the proliferation of various types of cells, including endothelial cells, fibroblasts, keratinocytes, and macrophages [34, 35]. Failure of re-epithelization is an important hallmark of chronic non-healing wounds [36]. Furthermore, we investigated cell proliferation via immunohistochemical staining using anti-Ki-67 antibody. Nuclear antigen Ki-67 as the cell proliferation marker has been widely used to characterize proliferative cells [37]. The results showed that the percentage of Ki-67-positive cells in the WJMSCs + PF-127 + 400 μM SAP group (36.86 ± 3.00%) was higher than other groups (PBS, 2.98 ± 0.54%; SAP, 5.10 ± 0.61%; PF-127, 4.74 ± 1.45%; PF-127 + SAP, 5.66 ± 0.52%; WJMSCs, 8.00 ± 0.93%; WJMSCs + SAP, 11.51 ± 1.63%; WJMSCs + PF-127, 14.81 ± 1.92%) (Fig. 3a, d) (*p* < 0.001), suggesting re-epithelialization on cutaneous wound would begin.

Angiogenesis is essential for wound healing, and newly formed vasculature which delivers oxygen and nutrients to the wound site, facilitates granulation tissue growth during the process of the entire wound healing [38]. Reduced angiogenesis is a hallmark of non-healing diabetic foot ulcers [39]. Platelet endothelial cell adhesion molecule-1 (PECAM-1 or CD31) which is expressed in endothelial cells of vascular compartment plays vital role in angiogenesis [40]. In this study, we identified the neovascularization in diabetic wound via immunohistochemical staining with anti-CD31 antibody. The results showed that the number of CD31-positive cells in the WJMSCs + PF-127 + 400 μM SAP group (36.67 ± 2.73 per field) increased compared with other groups (PBS, 7.32 ± 0.33; SAP, 16.33 ± 1.76; PF-127, 14.67 ± 1.45; PF-127 + SAP, 17.67 ± 3.53; WJMSCs, 15.33 ± 1.20; WJMSCs + SAP, 24.33 ± 1.21; WJMSCs + PF-127, 25.33 ± 2.67 per field) (Fig. 3a, e) (*p* < 0.01). Taken together, WJMSC/PF127/SAP transplantation may facilitate macrophages transformation from M1 to M2, cell proliferation, and angiogenesis to promote diabetic wound healing.

### PF-127 plus SAP combination helps WJMSCs survival and engraftment in diabetic wound in vivo

**Fig. 4:**
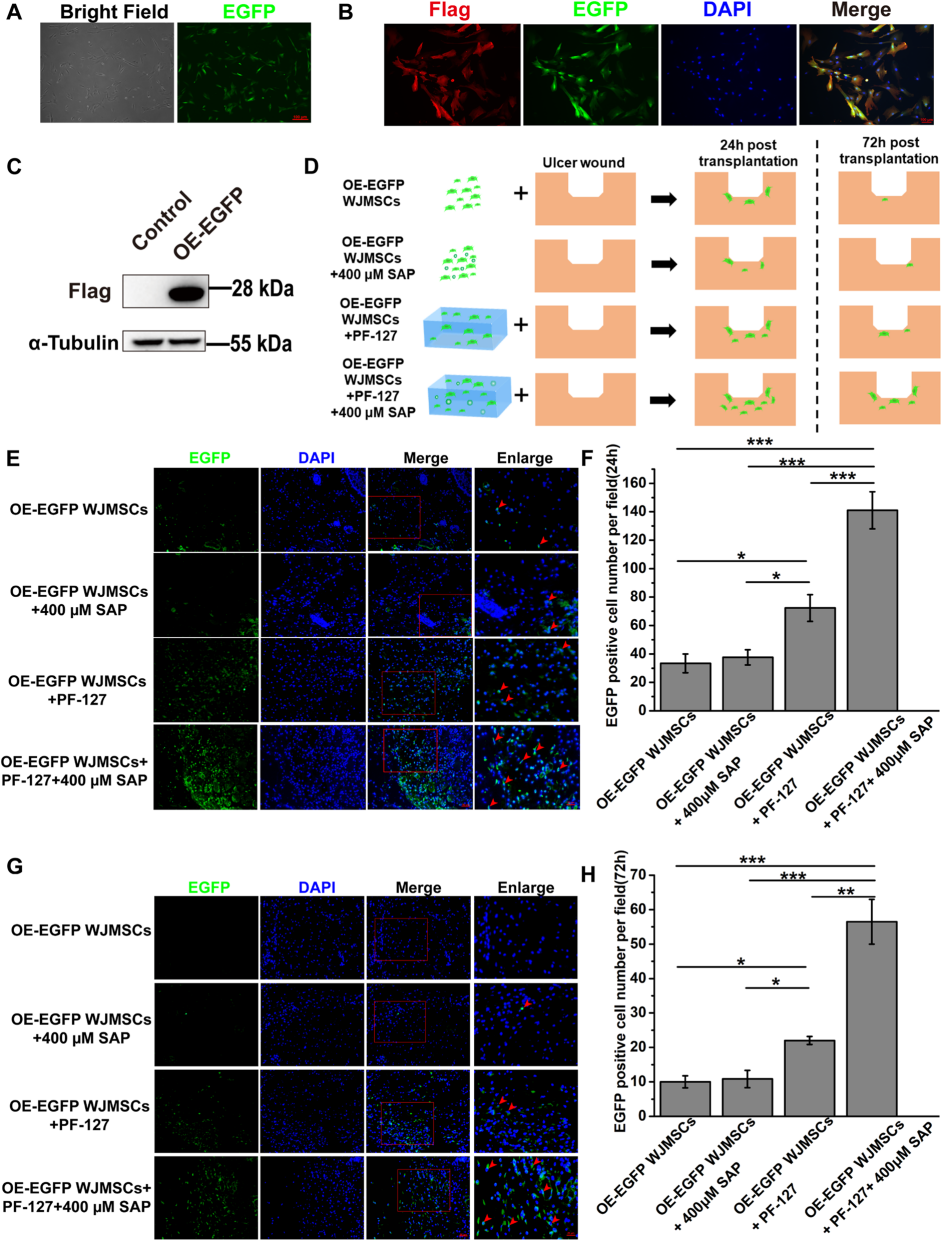
PF-127 plus SAP combination promotes engraftment of WJMSCs in diabetic wound.** a–c** Bright-field and fluorescence microscopy of WJMSCs stably expressing Flag-tagged EGFP was performed (A-B) and either western blotted to confirm EGFP expression with the indicated antibodies. The tubulin was shown as internal control. **d** Schematic of OE-EGFP WJMSCs migration by fluorescence assay at 24 h and 72 h after transplantation in ulcer wound. **e, f** At 24 h after transplantation, EGFP-overexpressing WJMSCs in different groups were detected (E) and the number of EGFP-positive WJMSCs was analyzed quantitatively in each group (F). Error bars represent mean ± SEM; n = 3 independent experiments. Significance was determined using one-way ANOVA. ***p < 0.001. Scale bar: 50 μm. Enlarge scale bar: 25 μm. **g, h** At 72 h after transplantation, EGFP-overexpressing WJMSCs in different groups were detected (**g**) and the number of EGFP-positive WJMSCs was analyzed quantitatively in each group (H). Error bars represent mean ± SEM; n = 3 independent experiments. Significance was determined using one-way ANOVA. ***p < 0.001. Scale bar: 50 μm. Enlarge scale bar: 25 μm

The therapeutic effectiveness of cell transplantation treatment is still limited by low cell retention, survivability, and engraftment [41]. To further investigate whether PF-127 and SAP enhanced WJMSCs retention and engraftment in diabetic wound, we constructed a stable EGFP expressing cell line (OE-EGFP WJMSCs) through lentivirus infection (Fig. 4a–c and Additional file 1: Fig. S2). We identified the cell existing and survival in the skin tissue by in situ immunofluorescence staining at 24 h and 72 h after transplantation, respectively (Fig. 4d). We found that the number of EGFP-positive WJMSCs in the dermis in the OE-EGFP WJMSCs + PF-127 + 400 μM SAP group (141.00 ± 13.01 per field) was higher than other three groups (OE-EGFP WJMSCs, 33.33 ± 6.57; OE-EGFP WJMSCs + 400 μM SAP, 37.67 ± 5.36; OE-EGFP WJMSCs + PF-127, 72.33 ± 9.39 per field) at 24 h post-transplantation (Fig. 4e, f). The number of surviving EGFP-positive WJMSCs in the OE-EGFP WJMSCs + PF-127 + 400 μM SAP group (56.50 ± 6.50 per field) also higher than other three groups (OE-EGFP WJMSCs, 10.00 ± 1.76; OE-EGFP WJMSCs + 400 μM SAP, 10.83 ± 2.52; OE-EGFP WJMSCs + PF-127, 22.00 ± 1.15 per field) at 72 h after transplantation (Fig. 4g, h). Immunofluorescence staining results also showed that the number of Flag-positive WJMSCs in OE-EGFP WJMSCs + PF-127 + 400 μM SAP group was higher than control groups (Additional file 1: Fig. S3). These results implied that PF-127 plus SAP not only prolongs the retention time of WJMSCs, but also improves the survival of transplanted WJMSCs.

### SAP supplementation decreases the WJMSCs apoptosis

**Fig. 5:**
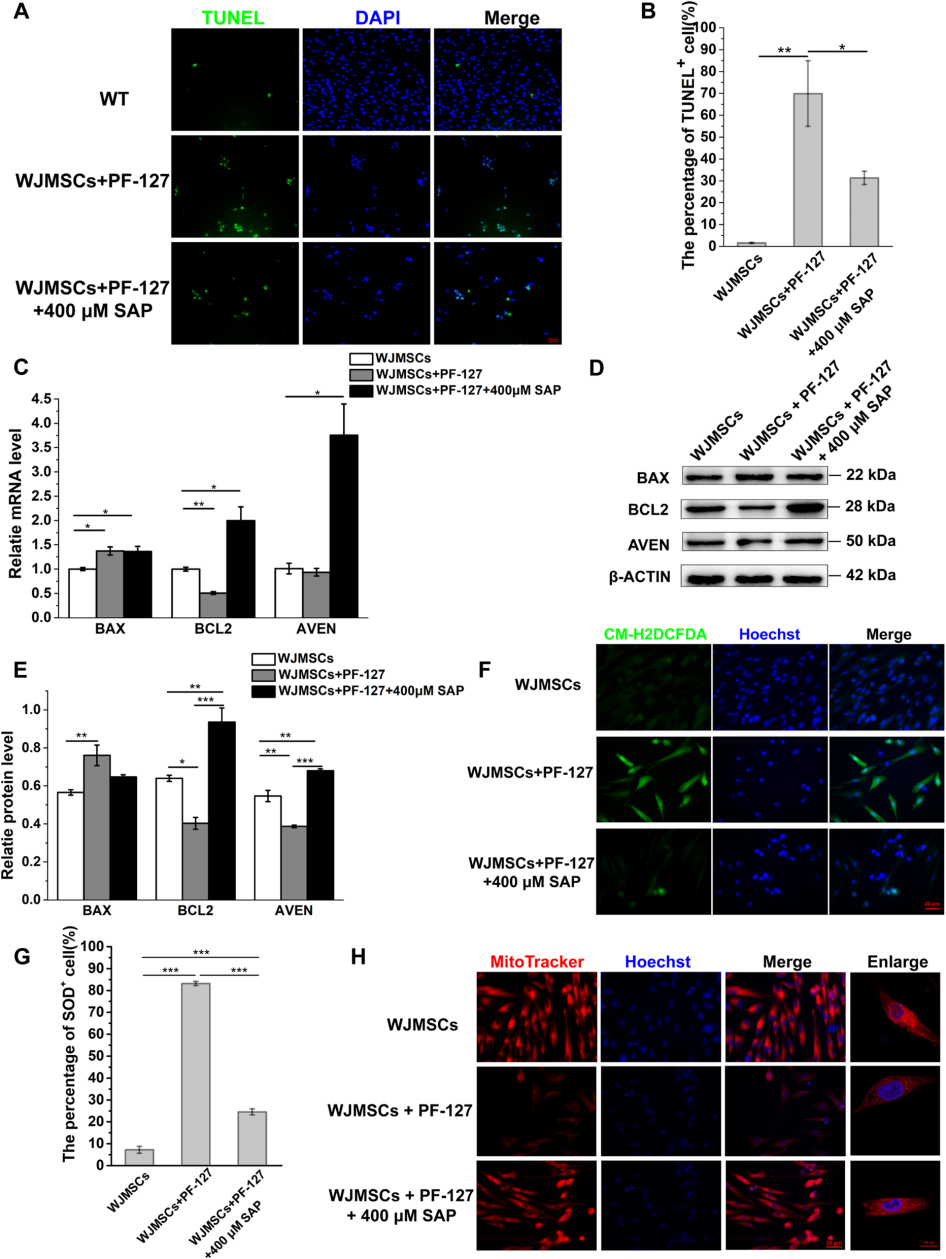
SAP supplementation alleviated the apoptosis of WJMSCs encapsulated with PF-127.** a** The cell apoptosis was detected by TUNEL immunofluorescence in different groups. TUNEL, green, represents apoptotic cell; DAPI, blue, represents cell nuclei. Scale bar, 100 μm. **b** The quantitative data of the percentage of TUNEL-positive cell per field were analyzed. Error bars represent mean ± SEM; n = 3 independent experiments. Significance was determined using one-way ANOVA. *p < 0.05, **p < 0.01. **c** The mRNA level of proapoptotic and antiapoptotic gene was measured by qRT-PCR in different groups. Error bars represent mean ± SEM; n = 3 independent experiments. Significance was determined using one-way ANOVA. *p < 0.05, **p < 0.01. **d** The protein level of proapoptotic and antiapoptotic gene was measured by western blot in different groups. **e** The quantitative data of protein level of different genes in different groups. Error bars represent mean ± SEM; n = 3 independent experiments. Significance was determined using one-way ANOVA. *p < 0.05, **p < 0.01, ***p < 0.001. **f** CMH2DCFAD staining was analyzed in different groups, CMH2DCFAD: green, represents the ROS fluorescence intensity; Hoechst, blue, represents cell nuclei. Scale bar: 20 μm. **g** The quantitative data of the percentage of CMH2DCFAD-positive cell per field were analyzed. Error bars represent mean ± SEM; n = 3 independent experiments. Significance was determined using one-way ANOVA. ***p < 0.001. **h** Mitochondrial location and distribution patterns were detected in different groups. Scale bar, 20 μm. Enlarge scale bar, 10 μm

Previous studies have been proved that PF-127 had a certain cytotoxicity to the encapsulated MSCs in vitro [42, 43]. Furthermore, we found that SAP supplementation (400 μM) in encapsulated WJMSCs with PF-127 can decrease the ratio of dead cells (Additional file 1: Fig. S4) [21]. We suspected that SAP may affect apoptosis to help WJMSCs survival in the early stage of transplantation. The cell apoptosis of WJMSCs via TUNEL immunofluorescence assay was performed in PF-127-encapsulated WJMSCs with or without SAP. In PF-127-encapsulated WJMSCs, the percentage of TUNEL-positive WJMSCs increased significantly from 1.56 ± 0.26% to 69.80 ± 14.97% (Fig. 5a, b). Once SAP was supplemented, the percentage of TUNEL-positive WJMSCs decreased to 31.29 ± 3.03% (Fig. 5a, b). In addition, the mRNA level of proapoptotic *BAX* gene increased slightly in the both PF-127-encapsulated WJMSCs and WJMSCs + PF-127 + 400 μM SAP group (*p* < 0.05) (Fig. 5c). Antiapoptotic *BCL2* gene and novel inhibitor of caspase activation *AVEV* gene increased significantly in the WJMSCs + PF-127 + 400 μM SAP group (*p* < 0.05), which is consistent with decreased TUNEL-positive cell in WJMSCs + PF-127 + 400 μM SAP group compared with PF-127-encapsulated WJMSCs (Fig. 5c). Consistent with the mRNA expression level (*p* < 0.01), western blot results showed that the protein level of *BAX* was increased in WJMSCs + PF-127 group, while slightly decreased in WJMSCs + PF-127 + 400 μM SAP group. Conversely, the protein level of *BCL2* and *AVEN* was both decreased the WJMSCs + PF-127 group, but supplementation of SAP increased the expression of *BCL2* and *AVEN* (*p* < 0.001) (Fig. 5d, e). These results demonstrated that supplementation of SAP rescued the apoptosis of WJMSCs which encapsulated with PF-127.

Mitochondria are multifunctional organelles which are responsible for energy production, cell apoptosis, and various biological processes. Dysfunctional mitochondria induce the cell apoptosis by releasing reactive oxygen species (ROS), mitochondrial signaling alteration, Ca^2+^ buffering, and apoptotic pathway activation [44, 45]. To explore the function of mitochondria in the WJMSCs embedded in the PF-127, we analyzed oxidative stress and mitochondrial damage by using CM-H2DCFDA fluorescence assay. The percentage of SOD-positive cell in WJMSCs + PF-127 group was 83.12 ± 0.97%, which was much higher than the WJMSCs group (7.18 ± 1.58%) (*p* < 0.001). After SAP supplementation, the percentage was significantly decreased to 24.47 ± 1.40% (*p* < 0.001) (Fig. 5f, g). MitoTracker as a fluorescent dye can stain mitochondria in living cells, and its accumulation depends on membrane potential and has been widely used for mitochondria tracking [46, 47]. We found that MitoTracker loading in WJMSCs + PF-127 group decreased, while SAP supplementation increased MitoTracker loading in WJMSCs + PF-127 + SAP group (Fig. 5h).

**Fig. 6:**
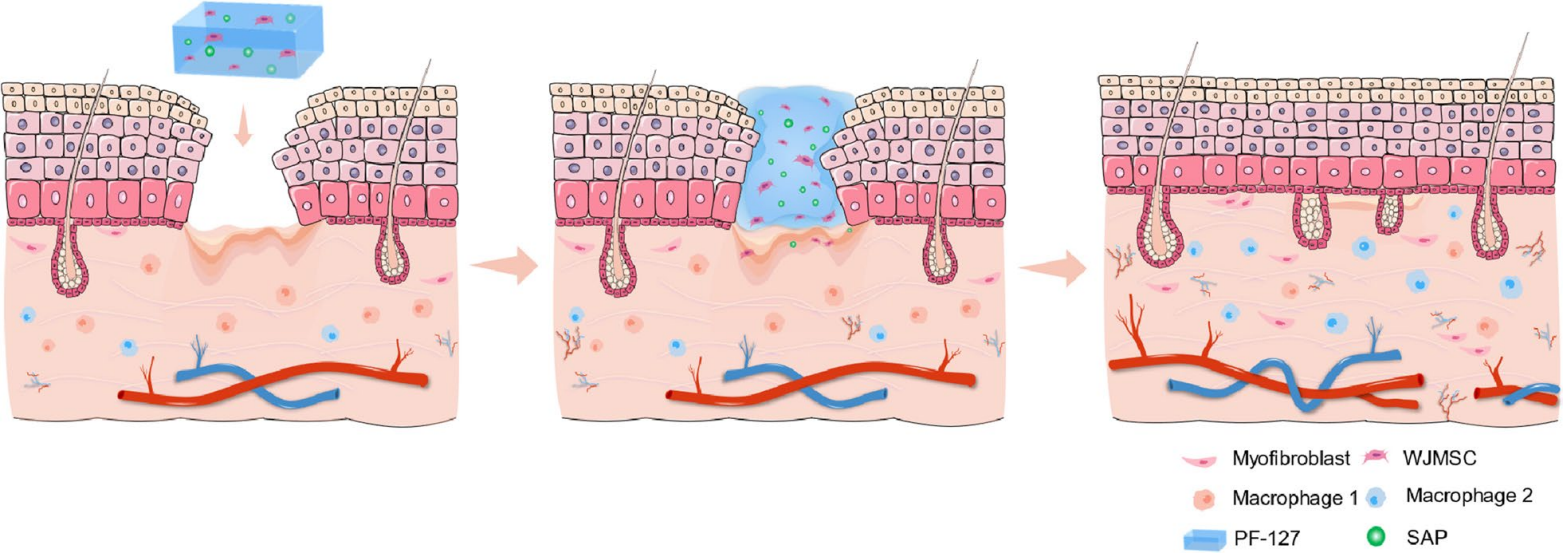
Working model of WJMSC + PF127 + SAP transplantation in diabetic rats. Transplantation of WJMSCs encapsulated with PF-127 and SAP can improve the diabetic wound healing and accelerate the macrophage transformation, cell proliferation, and neovascularization in the diabetic rats

Altogether, these results suggest that supplementation of SAP decreases the apoptosis ratio of WJMSCs embedded in PF-127 through alleviating oxidative stress and mitochondrial damage. These results combined support a notion that WJMSC/PF127/SAP transplantation promoting the wound healing of diabetic ulcer may improve macrophage transformation, cell survival, and neovascularization in the diabetic wound (Fig. 6).

## Discussion

At present, patients with DFU are still bearing a high risk of amputation and high costs of treatment and care. The transplantation of MSCs which have immunomodulatory and regenerative effects may be a cost-effective manner for non-healing wounds, especially for patients with DFU. However, hyperglycemia in DFU severely reduces the therapeutic effect of MSCs, which affect cell survival and engraftment [11, 12]. During MSCs transplantation, how to make MSCs-derived cell therapy effective is an important problem. In our study, we found that PF-127 plus SAP combination facilitates WJMSCs-mediated diabetic cutaneous ulcer wound healing in rat. The strategy increased the survival and viability of transplanted WJMSCs and promoted the macrophage transformation and angiogenesis in vivo. This study will provide a new MSCs-derived therapeutic strategy for diabetic wound healing.

Transplantation of WJMSCs encapsulated with PF-127/SAP combination promotes immunoregulation at diabetic wound. In response to chronic wounds, activated M1 pro-inflammatory macrophages are responsible for combating infections, whereas activated M2 macrophages are connected with tissue remodeling [30, 48]. In the DFU patients, the inflammatory response is perturbed and ineffective [49], showing M1 macrophages are accumulated and cannot convert to M2 macrophages owing to environmental stimuli [50]. In our study, transplantation of WJMSCs embedded in the PF-127 plus SAP combination decreased the number of CD86^+^ M1 macrophage while increased the number of CD163^+^ M2 macrophage in the diabetic wound. These results implied that the strategy ameliorated ineffective inflammatory response and advanced anti-inflammation transformation in diabetic rats.

Generally, cutaneous wound repair process can be divided into four overlapping phase, including coagulation, inflammation, granulation tissue formation, and remodeling or scar formation [51]. Granulation formation is the last step in the proliferation phase which concerted action of the proliferation and migration of various kind of cells, including endothelial cells, fibroblasts, keratinocytes, and macrophages [51, 52]. In chronic non-healing wounds of DFU, hyperglycemia severely blocked the proliferation, migration, homing, secretion of fibroblasts and endothelial cells, and disturbing granulation tissue formation [36, 53]. In our study, increased Ki-67-positive proliferating cells in diabetic wound after WJMSCs + PF-127 + SAP transplantation maybe help granulation tissue formation. In addition, SAP supplementation decreased the apoptosis and the ROS level of WJMSCs embedded in PF-127. Besides, PF-127 plus SAP combination accumulated more EGFP-positive WJMSCs in the dermis at both 24 h and 72 h after transplantation. PF-127 plus SAP combination not only prolonged the retention time of WJMSCs at ulcer wound bed, but also increased the survival and viability of transplanted WJMSCs. These results combined support a notion that WJMSCs + PF-127 + SAP can resist the stimulation of the deep chronic wounds and local PF127 toxicity to improve the quality of WJMSCs during granulation tissue regeneration in diabetic rats.

Furthermore, the restoration of the vascular system during wound healing is a complex process, which is crucial for the restoration of blood flow and transportation of nutrients to the injured site [51, 54]. Accumulating evidences have pointed out that MSCs stimulates wound healing by promoting angiogenesis by releasing angiogenic cytokines in diabetic ulcer [55, 56]. WJMSCs not only show multipotent differentiation toward angiogenic cell, such as vascular smooth muscle cells, endothelial cells, and other cell types, but also can secrete a variety of regulatory factors to promoting angiogenesis, inhibiting apoptosis, and modulating immunoreaction [57]. In our study, more CD31-positive cells were detected in the WJMSCs + PF-127 + 400 μM SAP group compared to other groups, demonstrating that the transplantation of WJMSCs encapsulated with PF-127 and SAP improved the angiogenesis in the diabetic wound.

## Conclusion

Collectively, our study reveals a novel and effective system to delivery WJMSCs to treat the diabetic wound. PF-127 plus SAP combination facilitates WJMSCs-mediated diabetic wound healing in rat through promoting the macrophage transformation, cell proliferation, and neovascularization. Our findings may potentially provide a helpful therapeutic strategy for patients with diabetic cutaneous ulcer.

## Supplementary Information

**Additional file 1.** Wharton’s jelly mesenchymal stem cells embedded in PF-127 hydrogel plus sodium ascorbyl phosphate combination promote diabetic wound healing in type 2 diabetic rat.

## Data Availability

All data generated and/or analyzed in this study are included in this published article.

## Abbreviations

SD rat: Sprague Dawley rat
ANOVA: Analysis of variance
CCK8: Cell Counting Kit-8
CD: Cluster of differentiation
DAPI: Diamidinophenylindole
DMEM-F12: Dulbecco’s modified Eagle’s medium-F12
EDTA: Ethylenediaminetetraacetic acid
EdU: Ethynyldeoxyuridine
PEI: Cationic polymer polyethylenimine
FBS: Fetal bovine serum
PBS: Phosphate buffer saline
PE: Phycoerythrin
TUNEL: Terminal deoxynucleotidyl transferase-mediated dUTP nick end-labeling
PF-127: Pluronic F-127
PFA: Paraformaldehyde
ROS: Reactive oxygen species
SAP: Sodium ascorbyl phosphate
UC: Umbilical cord
WJMSCs: Wharton’s jelly mesenchymal stem cells
H&E: Hematoxylin and eosin
APAF1: Apoptotic peptidase activating factor 1
AVEN: Apoptosis and caspase activation inhibitor
BCL2: BCL2 apoptosis regulator
BAX: BCL2-associated X, apoptosis regulator
