# Supplementary material for: Wharton’s jelly mesenchymal stem cells embedded in PF-127 hydrogel plus sodium ascorbyl phosphate combination promote diabetic wound healing in type 2 diabetic rat
Source: Stem Cell Res Ther. 2021 Oct 30;12:559. doi: 10.1186/s13287-021-02626-w (PMC8557497; doi:10.1186/s13287-021-02626-w)
Supplement: Supplementary file 1 — Additional file 1. Wharton’s jelly mesenchymal stem cells embedded in PF-127 hydrogel plus sodium ascorbyl phosphate combination promote diabetic wound healing in type 2 diabetic rat. [file 13287_2021_2626_MOESM1_ESM.docx]

**Supplementary Figure S1.**

**A**. Body weight of SD rats were measured every week during the model establishment of diabetic cutaneous ulcer, n=8; **B**. Fasting blood sugar of SD rats were measured before STZ injection and at 3 and 7 days after STZ injection, respectively, n=8.


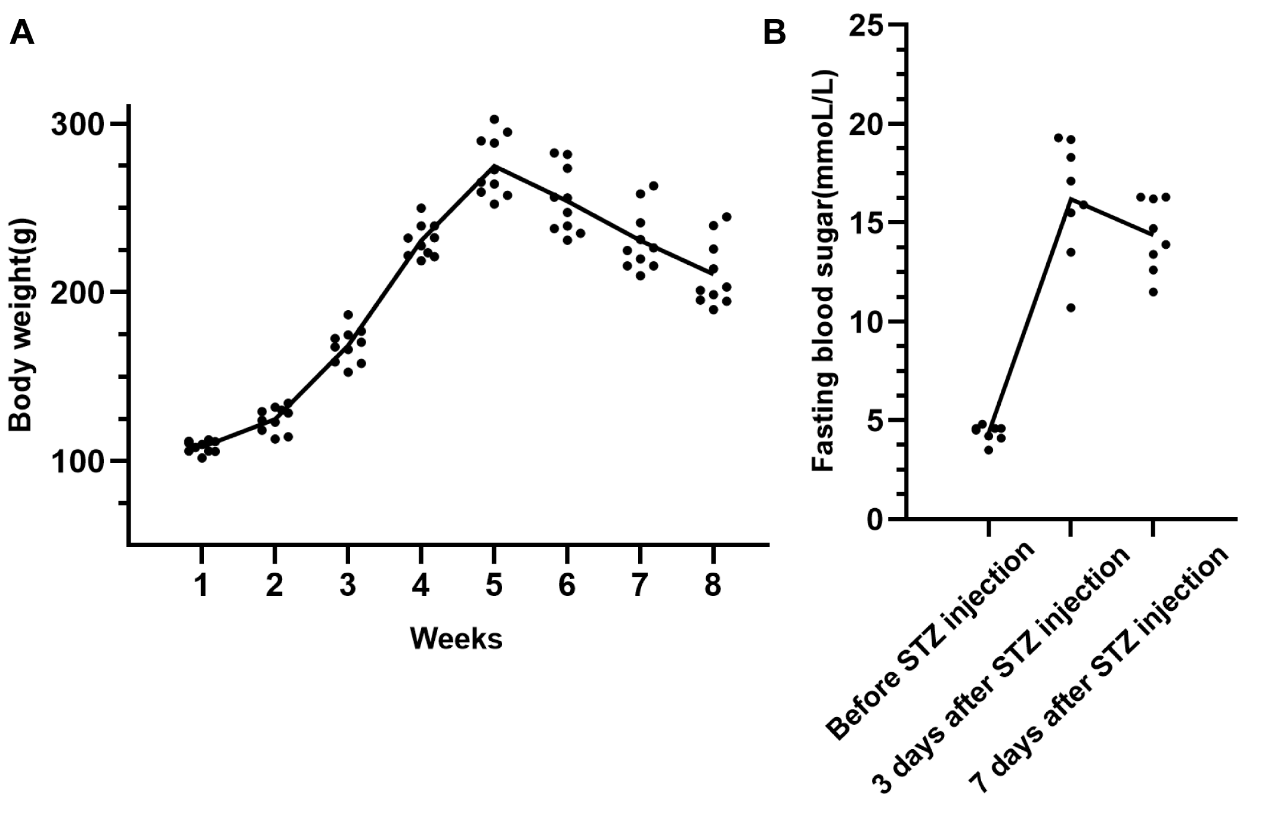


**Supplementary Figure S2.**

Lenti-CAG-EGFP-IRES-Puro plasmid for OE-EGFP WJMSCs stable line is constructed.


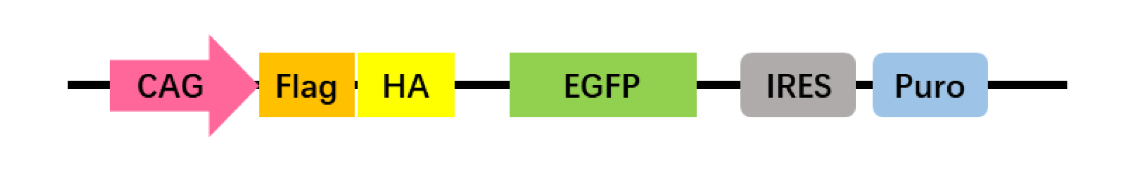


**Supplementary Figure S3. Immunofluorescence staining identified the expression of Flag in diabetic skin tissues.**

**A.** Representative immunofluorescence staining images of Flag in different groups skin tissues after OE-EGFP WJMSCs transplantation at 24 h. Scale bar, 50 μm. **B.** Representative immunofluorescence staining images of Flag in different groups skin tissues after OE-EGFP WJMSCs transplantation at 24 h. Scale bar, 50 μm.


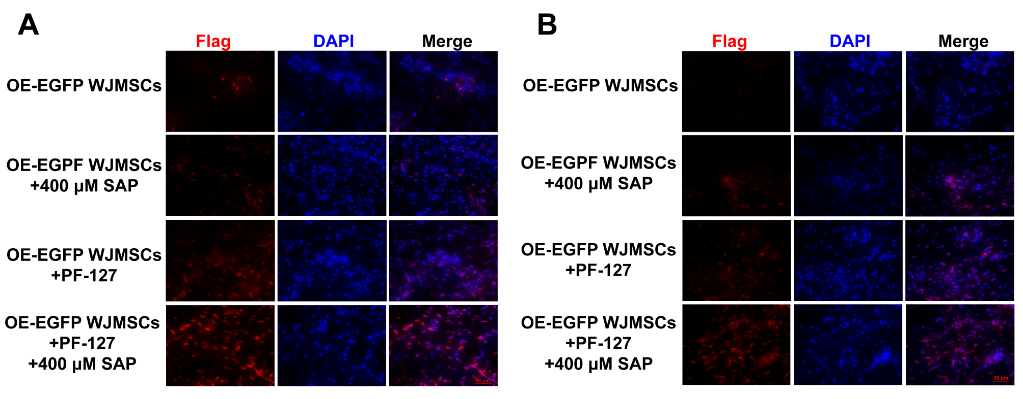


**Supplementary Figure S4.** SAP supplementation alleviates the cytotoxicity of PF-127 without affects the proliferation of WJMSCs.

**A**. The survival of WJMSCs was detected using Live/Dead^TM^ Cell Imaging Kit. Calcein staining (green) represents live cells, while PI staining (red) represents dead cells. Scale bar, 200 μm. **B**. The percentage of dead cells per field was analyzed and the quantitative histograms were showed. Error bars represent mean±SEM; n=3 independent experiments. Significance was determined using one-way ANOVA. **p < 0.01, ***p < 0.001. **C**. The cell viability of WJMSCs was detected by CCK-8 and the absorbance at 450 nm was measured by microplate reader. Error bars represent mean±SEM; n=3 independent experiments. Significance was determined using one-way ANOVA. ***p < 0.001. **D**. EdU staining of WJMSCs in different groups was showed. Red: EdU positive cells; Blue: DAPI. **E**. The percentage of EdU positive cells per field was analyzed and the quantitative histograms were showed. Error bars represent mean±SEM; n=3 independent experiments. Significance was determined using one-way ANOVA. **p < 0.01, ***p < 0.001.

**
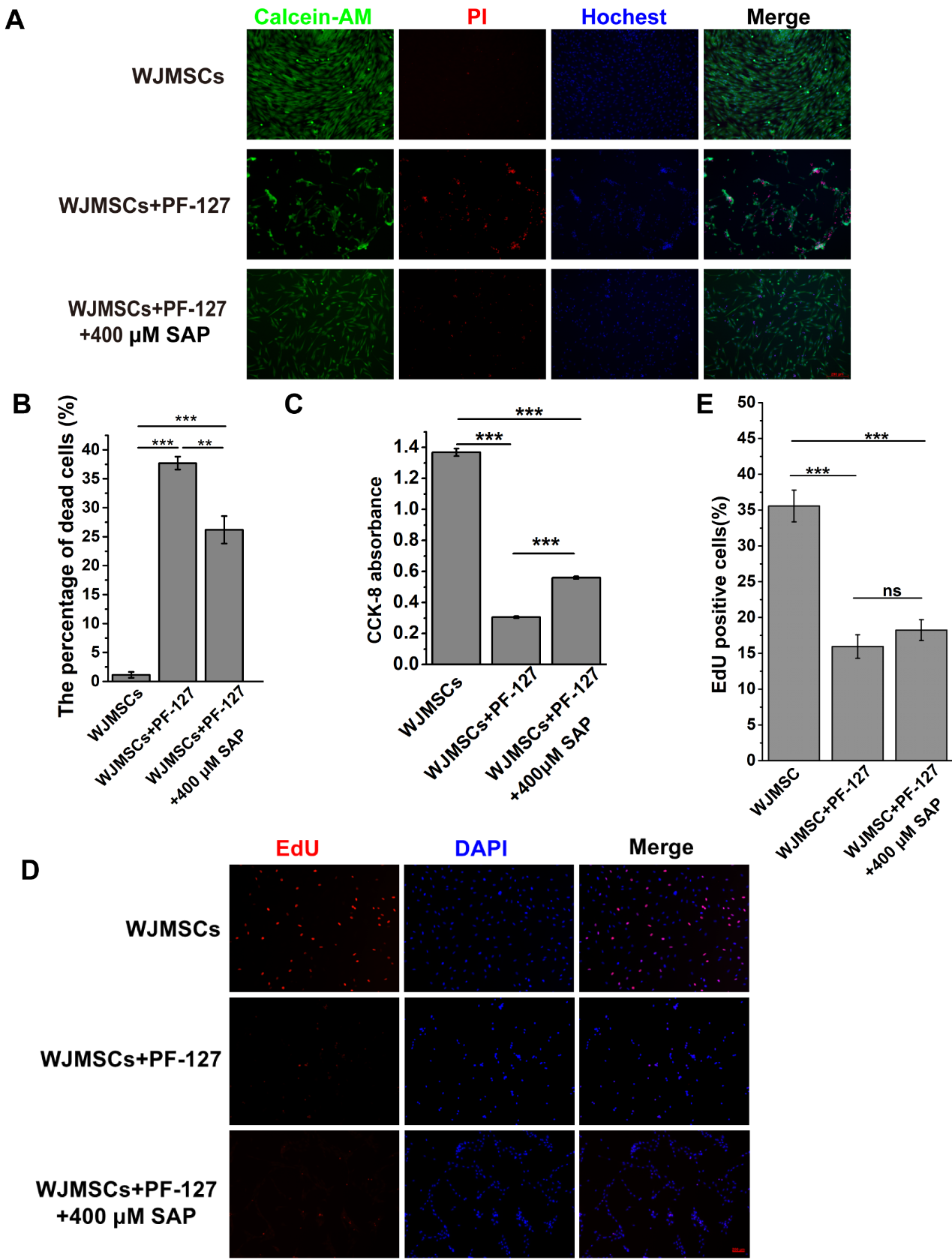
**

**Supplementary Table S1. Primer sequences used for qRT-PCR**

| Gene Name | Sequences of primers (5’-3’) | |
| --- | --- | --- |
|  | Forwards | Reverse |
| BCL2 | CCACCAAGAAAGCAGGAAACC | GCAGGATAGCAGCACAGGATT |
| BAX | TCCTGCACCTGTTACGGTTT | TGCTTCCAATTACTTTAATCCTTTTT |
| AVEN | CTCTGCCTCCGACTCAACG | TGCTTCCAATTACTTTAATCCTTTTT |
| β-ACTIN | GGCACCCAGCACAATGAA | TAGAAGCATTTGCGGTGG |

**Supplementary Table S2. Information of antibodies used in this study**

Primary antibodies used in the study

| Primary antibody | Type | Company | Catalog No. | Dilution rate |
| --- | --- | --- | --- | --- |
| BAX | Monoclonal | SANTA CRUZ | sc-7382 | 1:1000 |
| BCL-2 | Monoclonal | SANTA CRUZ | sc-23959 | 1:1000 |
| AVEN | Monoclonal | Abcam | ab133285 | 1:1000 |
| β-actin | Monoclonal | ABclonal | AC026 | 1:50000 |

Secondary antibody used in the study

| Secondary Antibody | Conjugate Used | | Company | | Catalog No. | Dilution rate |
| --- | --- | --- | --- | --- | --- | --- |
| Goat Anti-mouse IgG | | HRP | | Cell Signaling Technology | 7076 | 1:3000 |
| Goat Anti-rabbit IgG | | HRP | | Cell Signaling Technology | 7074 | 1:3000 |
